# *Tanacetum vulgare* as a Bioindicator of Trace-Metal Contamination: A Study of a Naturally Colonized Open-Pit Lignite Mine

**DOI:** 10.1007/s00244-013-9922-4

**Published:** 2013-06-08

**Authors:** Mateusz Jasion, Aleksandra Samecka-Cymerman, Krzysztof Kolon, Alexander J. Kempers

**Affiliations:** 1 Department of Ecology, Biogeochemistry and Environmental Protection, Wrocław University, ul. Kanonia 6/8, 50-328 Wroclaw, Poland; 2 Department of Environmental Science, Institute for Water and Wetland Research, Radboud University Nijmegen, Heyendaalseweg 135, 6525 AJ Nijmegen, The Netherlands

## Abstract

We investigated the possibility of use of *Tanacetum vulgare* (tansy) as an ecological indicator of metal concentration in a naturally colonized open-pit lignite mine in Bełchatów (Poland). *Tanacetum vulgare* is the only species growing abundantly and spontaneously in the lignite mine waste dumps. Metal concentrations in roots, stems, leaves, flowers, and soil were measured in dump sites differing in type and time of reclamation and therefore differing in pollution levels. *Tanacetum vulgare* appeared to be an accumulator of chromium and iron in roots, whereas highest concentrations of manganese and zinc were found in leaves. A high bioaccumulation factor for cadmium (Cd) was observed in dumps and control sites, indicating that even small amounts of Cd in the environment may result in significant uptake by the plant. The lowest concentrations of metals were found in plants from sites situated on dumps reclaimed with argillaceous limestone.

Large-scale open-pit mining of lignite leads to degradation of land and generation of waste areas that adversely affect the environment. The mining industry is a major source of soil contamination with trace elements or other pollutants influencing the ecosystem development (Maiti 2007). In Poland, lignite can be found among other coal ores in deposits in Bełchatów (central Poland), the largest of its kind in Poland and one of the largest mining sites in Europe. The production capacity since 1975 has been ≤33 million tons/year to supply fuel to a neighboring power plant (5,354-MW power capacity). Mining and combustion of lignite has led to the production of high emissions, including metals accumulated in soils around this urban and industrial area (Zier et al. 1999). Although overall improvements in pollution control decreased the emissions substantially, the area is still a source of atmospheric xenobiotics (Klose et al. 2003). The mining area is recognized as a hot spot related to metal pollution and strong soil acidification (pH 1.8–3.8) near dump sites. Open-pit coal mining alters landscapes, particularly through the excavation of spoil material and its deposition at dumps, which results in massive destruction of soil and soil biota (Holec and Frouz 2005). Approximately 100 million m^3^ of lignite waste has been excavated and dumped per year. These overburden dumps typically contain high amounts of all kinds of phytotoxic substrates and other constituents that lead to inorganic pollution with high metal concentrations in percolating water in soil and subsoil (Hüttl and Weber 2001; Sun et al. 2011). There is a large demand for reclamation of these acidic, nutrient-poor phytotoxic substrates (Gzyl 1999; Hüttl and Weber 2001). Neutralization and reclamation has led to an improvement of the soil pH to 6.0–6.5, and at some locations plants are now colonizing the dumps (Gzyl 1999). In the Belchatów site, some lignite waste dumps were reclaimed with application of lignite fly ashes. Generally fine lignite ash is applied to cover the dumps (Wisniewski 2001). Such fly ash has been regarded as a problematic solid because it leads to land degradation and contamination (Pandey and Singh 2010).

In this investigation we compared metal concentrations in soils, leaves, shoots, flowers, and roots of *Tanacetum vulgare* collected in the various types of dumps. We selected *T. vulgare* because it is a common and widespread plant with a high degree of adaptability and grows spontaneously in lignite dumps.

*Tanacetum vulgare*’s wide distribution indicates high ecological plasticity in different environmental conditions (Stevović et al. 2010, 2011). Therefore, it was of interest to investigate the bioaccumulation abilities of this species. In addition, monitoring of metal concentrations is necessary in this type of environment (Sun et al. 2011). The aim of this work was to study the impact of different reclamation methods on the availability of metal levels in waste dumps and the accumulation in *T. vulgare*. The tested hypotheses are (1) whether ubiquitous *T. vulgare* may be a suitable bioindicator of metal pollution for lignite mine waste; and (2) whether greater concentration of accumulated metals in roots rather than stems, leaves, or flowers maybe a form of plant strategy of protection of photosynthetic tissues of this species.

## Materials and Methods

### Sampling Design

**Fig. 1:**
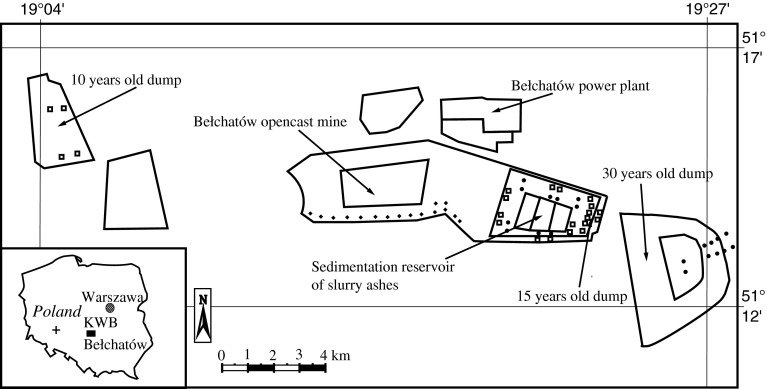
Location of the sampling sites of soil and plant material in the Bełchatów mine (KWB). Sites on an approximately 30-year-old dump, sites situated closest to the ashes disposal reservoir (*filled circle*), and sites situated on the slope of the upper part of the open mine (*closed diamonds*), sites from the approximately 15-year-old dump, sites from the still active 2-year-old dump, and sites from the 10-year-old dump (*open squares*), control site (*plus sign*)

In the Bełchatów mine (Fig. 1) a total of 59 sampling sites in 5 areas were selected: 10 sites in an approximately 30-year-old dump of ashes covered by overburden, 20 sites (situated on the east side of a pond still serving as a sedimentation reservoir for ash slurry) in an approximately 15-year-old dump covered with overburden and argillaceous limestone; 8 sites on the west side of the sedimentation reservoir on a 2-year-old dump of ashes mixed with overburdened and argillaceous limestone where dumping is still under way; 16 sites on the slopes of the upper part of an open-pit mine pit mine; and 4 sites located on a 10-year-old dump consisting of overburden. An unpolluted control site was selected 180 km west from Bełchatów within a forest clearing in the vicinity of Trzebnica (N:51°20′; E:17°8′), in southwest Poland, with a similar soil and climate. All collected plants colonizing the study area were found growing naturally in open places and thus were not affected by canopy throughfall. At each site, 5 root samples together with above-ground biomass (divided later into leaves, stems, and flowers) were collected randomly within a 25 × 25-m^2^. Soil samples from a depth of 0–5 cm were also taken from each grid. Each plant and soil sample consisted of a mixture of three subsamples. The total number of plant (roots, stems, leaves, and flowers) and soil samples was 295 (5 samples at each of 59 sites). The investigation was repeated in 2011 and 2012. There was no significant difference between data from both years.

### Soil and Plant Analysis

Fresh soil samples were used for the determination of pH_H20_ and pH_KCl_ potentiometrically (Model: Hanna HI991300, Hanna Instruments Inc., Wooinsocket (RI), USA). Soil and plant samples were dried at 50 °C to a constant weight. Soil samples were homogenized with mortar and pestle after coarse material had been removed using a 2-mm sieve. Plant samples were homogenized to a fine powder in an IKA (Labortechnik M20) laboratory mill. Dried soil and plant samples (300 mg in triplicate) were digested with 3 mL HNO_3_ (ultra pure [65 %]) and 2 mL HCLO_4_ (ultra pure [70 %]) in a microwave oven (Model: CEM Mars 5). After dilution to 50 mL, the soil and plant digests were analyzed for iron (Fe), manganese (Mn), and zinc (Zn) using flame atomic absorption spectrophotometry (AAS) and for cadmium (Cd), cobalt (Co), chromium (Cr), copper (Cu), nickel (Ni), and lead (Pb) using ETAAS (Electrothermal Atomic Absorption Spectrometry) with Graphite Furnace GF3000 [Model: AVANTA PM AAS (GBC Scientific Equipment)]. All elements were assayed against standards (AAS standard solution from Sigma Chemical) and blanks containing the same matrix as the samples and were subjected to the same procedure. All results for the plants were calculated on a dry-weight basis. The accuracy of the methods applied for the determination of the elements in plants and soil samples was checked against certified reference materials. In the present study, DC73348 LGC standards of bush branches and leaves and RTH 907 Dutch Anthropogenic Soil (Wageningen Evaluating Programs for Analytical Laboratories) were used as certified reference materials. The coefficient of variance (CV) was calculated for the determined concentrations of elements in the reference materials (Table 1).

**Table 1:** Analysis of certified reference material

Element	Bush branches and leaves (C73348 LGC)				Dutch anthropogenic soil (RTH907)			
	Certified (mg kg^−1^)	Found	Recovery (%)	CV	Certified (mg kg^−1^)	Found	Recovery (%)	CV
Cd	0.140 ± 0.06	0.139 ± 0.006	99.29	4.3	2.18 ± 0.33	2.20 ± 0.08	100.92	3.6
Co	0.39 ± 0.05	0.38 ± 0.01	97.52	2.6	9.09 ± 1.55	8.99 ± 0.17	98.90	1.9
Cr	2.30 ± 0.30	2.29 ± 0.05	99.57	2.2	48.60 ± 6.64	53.09 ± 1.02	109.24	1.9
Cu	5.20 ± 0.50	5.06 ± 0.12	97.31	2.4	121.00 ± 12.00	119.94 ± 1.53	99.12	1.3
Fe	1,020 ± 67.00	1,051 ± 21.00	103.04	2.0	16.60 ± 2.13	17.27 ± 0.55	104.04	3.2
Mn	58.00 ± 6.00	58.37 ± 1.33	100.63	2.3	506 ± 47.00	531 ± 13.00	104.94	2.4
Ni	1.70 ± 0.40	1.69 ± 0.06	99.41	3.6	27.90 ± 3.06	27.59 ± 1.02	98.89	3.7
Pb	7.10 ± 1.10	6.78 ± 0.27	95.49	4.0	318.00 ± 25.00	309.20 ± 6.50	97.23	2.1
Zn	20.60 ± 2.20	20.77 ± 0.36	100.83	1.7	714.00 ± 50.00	716.30 ± 9.90	100.32	1.4

### Statistical Analysis

Differences between sampling sites in terms of concentrations of elements in soil and plants were evaluated by one-way analysis of variance (ANOVA) on log-transformed data to obtain normal distribution of features according to Zar (1999). The normality of the analyzed features was checked using Shapiro–Wilk’s *W* test, and the homogeneity of variances was checked using Brown–Forsythe test. *Post hoc* LSD test was used to compare element concentrations between roots, stems, leaves, and flowers of *T. vulgare*. The matrix of concentrations of six metals (Co, Cu, Fe, Mn, Ni, and Zn) in *T. vulgare* from 59 sites was subjected to ordination to show any possible gradients of element levels by means of principal component and classification analysis (PCCA). A PCCA ordination plot of the plant samples and projection of the element concentrations in *T. vulgare* on the factor plane, according to Legendre and Legendre (1998), gives information about similarities between samples and shows correlations between the original variables and the first two factors. Cd, Cr, and Pb concentrations were added as supplementary variables because the PCCA showed that these elements decreased variance much more than the other elements. The PCs were then computed using only the active variables. The supplementary variable was later projected onto the vector subspace generated by the factors thus computed. Conclusion were drawn about the Cd, Cr, and Pb concentration variables, even though they were not included in the analysis. All calculations were performed using STATISTICA 10 (2011) from Statsoft, StatSoft Inc., Tulsa, OK, USA.

## Results and Discussion

**Fig. 2:**
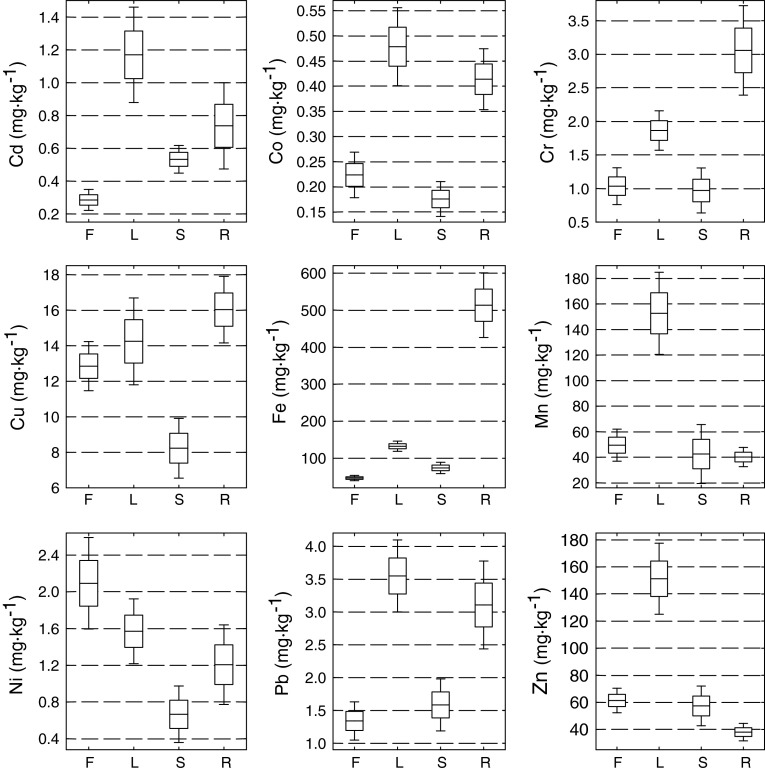
(*Dashes*) mean, (*open square*) SE, and (*brackets*) confidence interval of concentrations of elements in flowers (*F*), leaves (*L*), stems (*S*), and roots (*R*) of *T. vulgare*

The pH and concentration ranges of elements in the soil of the 58 Belchatów sites and concentrations of elements in leaves, stems, flowers, and roots of *T. vulgare* (excluding those from the control site) are shown in Table 2 and Fig. 2. Statistical analysis (ANOVA, *P* < 0.05) showed significant differences among element concentrations in the soil and plant samples. Most soil samples (Table 2) exceeded the values typical for clean soils of *T. vulgare* sites established by Stevović et al. (2010, 2011). Metal concentrations in the soil of the control site did not exceed the limits for unpolluted soils (Kabata-Pendias 2001) and was found to be significantly lower (Student *t* test, *P* < 0.05) than in the Bełchatów samples. The above-mentioned results indicate that metal contamination of the soil is probably due to a release of metals from the lignite overburden waste. Compared with *T. vulgare* investigated by Stevović et al. (2010, 2011) in a nonanthropogenic area, the species from Bełchatów accumulated more Cd, Cr, Ni, and Pb in leaves, stems, and roots.

**Table 2:** Minimum and maximum values, mean and SD of pH, and concentrations of elements (mg kg^−1^) in soil of *T. vulgare*

Element	Bełchatów				Unpolluted^a^
	Minimum	Maximum	Mean	SD	
pH_H20_	4.0	8.4	7.5	0.8	
pH_KCl_	3.9	8.3	7.1	1.0	
Cd	0.01	0.2	0.05	0.02	
Cr	3.7	16	7.6	2.2	<7.0
Co	0.5	11	4.8	0.6	
Cu	1.0	13	4.5	1.0	
Fe	840	7,340	3,950	700	<5,100
Mn	3.9	280	70	24	<600
Ni	1.4	34	16	5.0	<4.3
Pb	3.2	60	18	5.0	<9
Zn	1.7	41	13	3.0	

^a^Data in this column are from clean sites of this species investigated by Stevović et al. (2010, 2011)

To evaluate metal transfer from soil to roots, a bioaccumulation factor (BF) was calculated as a ratio of root to soil metal concentrations. The factor (in parentheses) can be ordered as follows: Cd (45) > Cu (12) > Zn (10) > Mn (3) > Cr (0.4) > Pb (0.3) > Co (0.2) > Fe (0.2) > Ni (0.1) for the Bełchatów plants and Cd (15) > Zn (7) > Cu (3) > Mn (0.7) > Pb (0.1) > Cr (0.06) > Co (0.08) > Fe (0.03) > Ni (0.02) for the control plants. *Tanacetum vulgare* growing in a relatively clean site accumulated high amounts of Cd. The BF examined by Bidar et al. (2009) for *Trifolium repens* and *Lolium perenne* confirmed that metals were preferentially accumulated in the roots in a similar order for the first two elements and that their transfer to shoots was limited. Metal transfer from roots to shoots (TF) was also evaluated as a ratio of metal concentrations in shoots to roots to provide an indication of internal metal transportation. This factor for *T. vulgare* indicated that metals accumulated by this species were mostly retained in the roots in the case of Cr (0.9) and Fe (0.4) for the Bełchatów plants and Cr (0.8) and Fe (0.2) for the control plants. The metals mostly transported from roots to shoots as shown by TFs > 1 were Mn (13), Ni (7.0), Zn (5.0), Cd (2.0), Pb (2.0), Cu (1.3) and Co (1.1) for the Bełchatów plants and Mn (6.0), Ni (6.0), Zn (3.0), Co, (2.0), Cu (2.0), Pb (2.0) and Cd (1.2) for the control plants. An extremely high BF and low TF for Cd in *T. vulgare* at the control site may be an indication for the potential of the plant for bioindication of this element (Fitz and Wenzel 2002; Naziri et al. 2011). However, although having BFs > 1, Co, Cr, Ni, and Pb were accumulated by *T. vulgare* in concentrations greater than typical for terrestrial plants from clean areas (Kabata-Pendias 2001). Padmavathiamma and Li (2012) had much lower TFs: Cu = 0.41–0.43, Mn = 0.78–0.92, Pb = 0.19–0.25, and Zn 0.69 = 0.78 for *L. perenne*, *Festuca rubra*, and *Poa pratensis* growing along highways in Canada. Significantly greater accumulation of Cr and Fe occurred in roots than in leaves, suggesting limited mobility and translocation of these metals once absorbed by *T. vulgare*. Sequestration of metals in roots enables plants to continue uninhibited growth, and it is an important mechanism of metal tolerance (Kachenko et al. 2007). These results are in agreement with Page (2002) and Olivares et al. (2009), i.e., that Cu, Mn, and Zn are essential plant micronutrients, and their uptake and allocation in plant organs, such as photosynthetic tissues, is high and active. Conversely, Cd and Pb are both nonessential to plant growth, and their translocation from roots to other plant organs is generally low (Page 2002 **).** According to Tyler (2004), many species accumulate nonessential metals mainly at the root level. *T. vulgare* from this investigation does not confirm the above-mentioned opinions because it transported Cd, Co, Ni, and Pb into leaves without visible effects on their morphology (necroses, chloroses). These features prove the ability of *T. vulgare* to bioaccumulate these metals during growth in a polluted environment. Cr and Fe concentrations in *T. vulgare* were the highest in roots, and Cd, Mn, and Zn concentrations were the highest in leaves (Fig. 2).

**Fig. 3:**
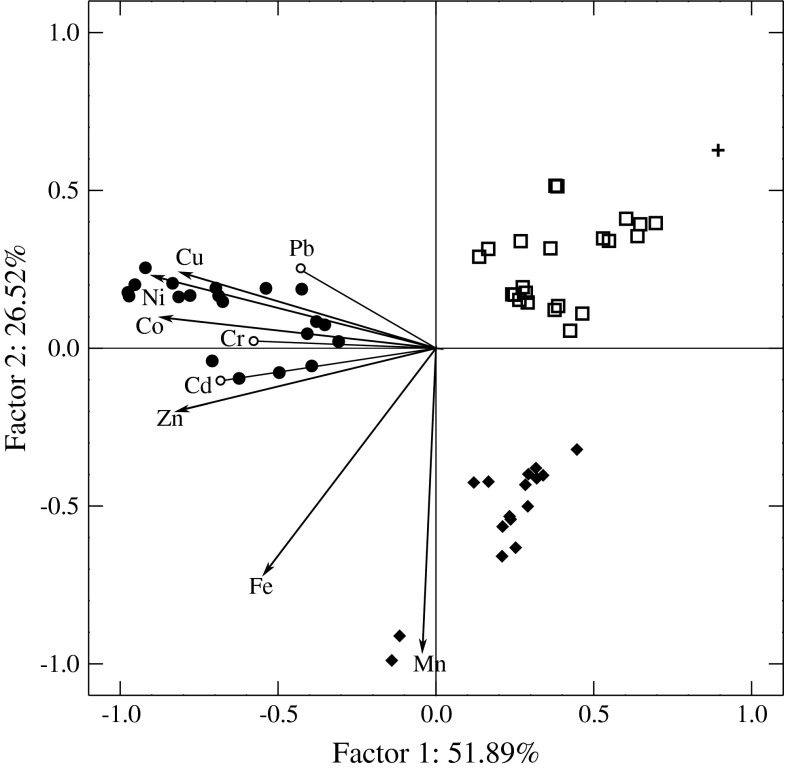
Ordination of the 59 sampling sites of *T. vulgare* by PCCA based on concentrations of 7 metals (Cd, Co, Cu, Fe, Mn, Ni, and Zn) and three supplementary metals (Cd, Cr, and Pb) and projection of the element concentrations in plants on the factor plane. Sites on an approximately 30-year-old dump, sites situated closest to the ashes disposal reservoir (*filled circle*), and sites situated on the slope of the upper part of the open mine (*closed diamonds*), sites from the approximately 15-year-old dump, sites from the still active 2-year-old dump, and sites from the 10-year-old dump (*open squares*),  control site (*plus sign*)

The PCCA ordination of the plants tested is shown in Fig. 3. The first principal component discriminates between *T. vulgare* growing in the approximately 30-year-old dump and at sites situated the closest to the ash disposal reservoir with the highest values of negative scores and all of the other sites with the highest values of positive scores. The second PC discriminates between *T. vulgare* at sites situated on the slope of the upper part of the open mine (negative scores) and sites at the approximately 15-year-old dump, sites at the still active 2-year-old dump, and sites at the 10-year-old dump (positive scores). The projection of the variables on the factor plane showed that *T. vulgare* sites with negative scores of factor one were characterized by the highest Co, Cu, Ni, and Zn as well as Cd, Cr, and Pb concentrations. *Tanacetum vulgare* from sites with negative scores of factor two was characterized by the highest Fe and Mn concentrations. *Tanacetum vulgare* from sites situated on the approximately 30-year-old dump and those situated the closest to the ash disposal reservoir had the highest Cd, Co, Cr, Cu, Ni, Pb, and Zn concentrations, which is in accordance with Sarris et al. (2009) and Suchara et al. (2011). According to these investigators, these metals are significantly accumulated in coal fly-ashes emitted by the lignite combustion industry. A greater level of fly ash incorporation results in heavy-metal pollution (Pandey and Singh 2010). Fly-ash particles, when dumped, cause a serious problem to the normal function of higher plants (Page 2002). In addition, the approximately 30-year-old dump was under the influence of a lignite power station, which at that time had no protection against the emitted xenobiotics. Sites with the highest Fe and Mn concentrations were situated on the slope of the open-pit mine in the vicinity of mine internal roads and pipes with drainage water from the mine. Probably the main source of Fe and Mn for *T. vulgare* at these sites was drainage water pumped under high pressure causing water spray being deposited as a red-brown layer on the surrounding surfaces. The water drained from the active open lignite mine contained increased levels of mainly Fe and Mn (Janiak 1992). The lowest metal concentrations were found in the 2- and approximately 15-year-old dumps reclaimed with argillaceous limestone. This is in agreement with Kabata-Pendias (2001), Zorrig et al. (2012), and Song et al. (2013), thus emphasizing the strong antagonistic and protective effect of Ca present in this rock against metals. According to Zorrig et al. (2012), fertilization with Ca appears to be a strategy for decreasing the risk of increased accumulation of metals by plants growing on contaminated soils.

## Conclusion

*Tanacetum vulgare* species has wide distribution indicating high ecological plasticity in different environmental conditions. An extremely high BF from soil to roots for Cd, not only at the open-pit lignite mine but also at the control site, which is an indication that even small amounts of Cd in the environment may cause significant uptake in the plant, makes this species a good bioindicator for this element. *Tanacetum vulgare* contained the highest Mn and Zn concentrations in leaves and was found to be a root accumulator of Cr and Fe. A greater concentration of accumulated metals in roots than in stems, leaves, or flowers is probably a plant strategy by this species to protect photosynthetic tissues. The lowest concentrations of metals in plants were found at sites situated in the dumps reclaimed with argillaceous limestone, which may indicate that Ca present in this rock seems to mitigate the severity of metals and decrease their toxicity. *Tanacetum vulgare* may be used as bioindicator of Cd, Mn, and Zn in areas of lignite industry.
